# Validating the Emergency Department Avoidability Classification (EDAC): A cluster randomized single-blinded agreement study

**DOI:** 10.1371/journal.pone.0297689

**Published:** 2024-01-23

**Authors:** Ryan P. Strum, Shawn Mondoux, Fabrice I. Mowbray, Lauren E. Griffith, Andrew Worster, Walter Tavares, Paul Miller, Komal Aryal, Ravi Sivakumaran, Andrew P. Costa

**Affiliations:** 1 Department of Health Research Methods, Evidence and Impact, McMaster University, Hamilton, Ontario, Canada; 2 Department of Medicine, Division of Emergency Medicine, McMaster University, Hamilton, Ontario, Canada; 3 Institute for Health Policy, Management and Evaluation, University of Toronto, Toronto, Ontario, Canada; 4 College of Nursing, Michigan State University, East Lansing, Michigan, United States of America; 5 McMaster Institute for Research and Aging, McMaster University, Hamilton, Ontario, Canada; 6 The Wilson Centre, University of Toronto, Toronto, Ontario, Canada; 7 Centre for Paramedic Education and Research, Hamilton Health Sciences, Hamilton, Ontario, Canada; 8 Health Information Management Department, St. Joseph’s Healthcare Hamilton, Hamilton, Ontario, Canada; Debre Markos University, ETHIOPIA

## Abstract

**Introduction:**

The Emergency Department Avoidability Classification (EDAC) retrospectively classifies emergency department (ED) visits that could have been safely managed in subacute primary care settings, but has not been validated against a criterion standard. A validated EDAC could enable accurate and reliable quantification of avoidable ED visits. We compared agreement between the EDAC and ED physician judgements to specify avoidable ED visits.

**Materials and methods:**

We conducted a cluster randomized, single-blinded agreement study in an academic hospital in Hamilton, Canada. ED visits between January 1, 2019, and December 31, 2019 were clustered based on EDAC classes and randomly sampled evenly. A total of 160 ED visit charts were randomly assigned to ten participating ED physicians at the academic hospital for evaluation. Physicians judged if the ED visit could have been managed appropriately in subacute primary care (an avoidable visit); each ED visit was evaluated by two physicians independently. We measured interrater agreement between physicians with a Cohen’s kappa and 95% confidence intervals (CI). We evaluated the correlation between the EDAC and physician judgements using a Spearman rank correlation and ordinal logistic regression with odds ratios (ORs) and 95% CIs. We examined the EDAC’s precision to identify avoidable ED visits using accuracy, sensitivity and specificity.

**Results:**

ED physicians agreed on 139 visits (86.9%) with a kappa of 0.69 (95% CI 0.59–0.79), indicating substantial agreement. Physicians judged 96.2% of ED visits classified as avoidable by the EDAC as suitable for management in subacute primary care. We found a high correlation between the EDAC and physician judgements (0.64), as well as a very strong association to classify avoidable ED visits (OR 80.0, 95% CI 17.1–374.9). The EDACs avoidable and potentially avoidable classes demonstrated strong accuracy to identify ED visits suitable for management in subacute care (82.8%, 95% CI 78.2–86.8).

**Discussion:**

The EDAC demonstrated strong evidence of criterion validity to classify avoidable ED visits. This classification has important potential for accurately monitoring trends in avoidable ED utilization, measuring proportions of ED volume attributed to avoidable visits and informing interventions intended at reducing ED use by patients who do not require emergency or life-saving healthcare.

## Introduction

Emergency departments (EDs) internationally have experienced significant increases in attendance over the past two decades from 2003 to 2023, with indications this trend will continue to escalate [1–4]. EDs are overcrowded, posing a significant challenge for the healthcare system [5, 6]. The consequences of overcrowding are wide-reaching, affecting both clinicians and patients (increased mortality [7–10], reduced quality of care [11], reduced treatment performance [11], increased medical errors [12], decreased compliance with treatment guidelines [13, 14]), ED staff (higher burnout and stress [15], lower job satisfaction [15]), and the broader healthcare system (increased per-visit costs due to extended lengths of stay [15, 16]). While patient boarding is the primary driver of overcrowding, it is a multifactorial issue influenced by poor patient flow, staffing shortages and rising patient volumes, particularly patients seeking ED care for non-emergent and minor health concerns [6, 17–19].

One strategy that may address this challenge is redirecting patients with specific non-emergent complaints and conditions to non-ED subacute care instead of the ED. Redirection interventions have yielded mixed results (i.e., electronic ED screening applications, secondary ED triage) [20, 21]. However, redirection has recently gained renewed focus as research continues to highlight managing non-emergent patients in the ED is associated with negative patient experiences, increased risk of hospital-acquired infections, staff burnout/emotional exhaustion and potentially higher healthcare costs [22–24]. A critical barrier to developing successful redirection interventions is the absence of an objective and consistent method for identifying patient cohorts suitable for these care models [20, 21, 25]. Previously published classifications for avoidable ED visits have shown to be unreliable and inconsistent classifiers due to a lack of validity evidence and consensus of classification criteria, yielding wide prevalence estimates that range from 5 to 90% [26, 27]. Their inaccuracies highlight the immediate need for an innovative epidemiological classification that is established with experimental validity to inform accurate intervention development.

The Emergency Department Avoidability Classification (EDAC) is an novel retrospective patient classifier that identifies ED visits that could have been appropriately managed and effectively treated in a subacute healthcare setting (an avoidable visit) [28]. The EDAC was developed through a collaborative effort involving expert emergency and primary physicians in Ontario, Canada, using a rigorous multi-stage and multicentered consensus process [29, 30]. The EDAC has demonstrated construct validity (i.e., the EDAC components represent the concept of avoidable ED visits) but has not been examined for evidence of criterion validity [31]. Successful validation would enable the EDAC to function as a trustworthy benchmark for policy stakeholders, epidemiologists and researchers to identify opportunities for modifying health policy, designing interventions to reduce avoidable ED visits, monitoring trends, and understanding gaps in community care that contribute to avoidable visits.

Our purpose was to examine the criterion validity of the EDAC to retrospectively classify ED visits that could have been managed in a subacute primary care against ED physician judgements. Our preliminary objective was to evaluate ED physician judgements as a criterion standard. Our main objective was to examine the correlation and association between the EDAC and ED physician judgments to specify avoidable ED visits. Our secondary objective aimed to assess the comparability of the mid-level class (potentially avoidable) with the avoidable and not avoidable EDAC classes.

## Materials and methods

### Study design

We conducted a cluster randomized, single-blinded agreement study. ED physicians were recruited from an academic hospital in Hamilton, Canada. Retrospective ED visits were categorized based on the EDAC into a three-cluster design (avoidable, potentially avoidable and not avoidable) and randomly sampled using a predetermined randomization protocol [32]. Physicians were randomly assigned ED charts from each study cluster evenly and judged whether the ED visit could have been safely managed in a subacute primary care setting. Physicians were blinded to the ED visits study cluster. We adhered to the study steps detailed in the study protocol [32].

### Selection of participants

ED physicians were eligible to participate in the study if they were (1) currently practicing, and (2) held a staff emergency physician position at the academic hospital. All physicians meeting the eligibility criteria were invited to participate in the study. An information letter and consent form were provided and all physicians were given the opportunity to review and ask questions prior to participating. Upon acceptance of participation, each physician signed and returned the study consent form. ED physicians provided demographic information about themselves, their length of clinical experience and medical training. Recruitment started September 7, 2022 and ended on October 18, 2022. We obtained informed consent from all twelve ED physicians invited to participate in the study.

### The Emergency Department Avoidability Classification

The EDAC is a patient classification that identifies retrospective ED visits that could have been appropriately and safely managed in a subacute primary care clinical setting [32]. The EDAC’s inclusion criteria were constructed in a multi-stage, multicentred consensus process involving emergency and primary care physicians [30, 33]. The consensus process assembled clinical and non-clinical characteristics readily available in administrative databases to retrospectively identify avoidable visits with a high specificity [30, 33]. The EDAC classifies ED visits as avoidable, potentially avoidable, and not avoidable. *Table 1* shows the EDAC classification logic: avoidable ED visits could have been safely managed in a subacute primary care centre, potentially avoidable ED visits could potentially have been managed in subacute primary care, and not avoidable ED visits could not be managed outside of the ED in subacute primary care.

**Table 1:** The Emergency Department Avoidability Classification (EDAC) criteria to identify avoidable, potentially avoidable and not avoidable ED visits.

EDAC Classes	Avoidable	Potentially Avoidable	Not Avoidable
**Definition**	ED visits that could have been managed by subacute primary care	ED visits that could potentially have been managed by subacute primary care	ED visits that could not have been managed by subacute primary care
**Age**, years	18–70		Not be classified as either Avoidable or Potentially Avoidable
**Triage Acuity**, CTAS	4 (Less Urgent) or 5 (Non-Urgent)	3 (Urgent)	
**Specialist Consultation in ED**	No		
**Main Physician Intervention**, CCI	Listed in S1 Table		
**ED Visit Outcome**	Discharged		

Note: CTAS = Canadian Triage and Acuity Scale, CCI = Canadian Classification of Health Interventions

### Selection of ED visits

Electronic ED visits at the academic hospital were eligible for study inclusion if the visit occurred between January 1, 2019, and December 31, 2019, all variables required to classify the visit using the EDAC were recorded in the chart (patient age, triage acuity, specialist consult completed, main physician intervention, ED visit outcome), and the patient did not leave against medical advice or without being assessed by an ED physician. We grouped included ED visits into three study clusters based on the EDAC classes: avoidable, potentially avoidable, and not avoidable. We randomly selected an equal quantity of ED visits from each study cluster to be included in the study, totaling 160 ED visits.

### Study completion

We randomly assigned a reasonably equal quantity of ED visits from each study cluster to each participating ED physician that totaled 20 ED visits (configuration of: seven, seven and six of the three study clusters). Overall, ten of the twelve ED physicians completed 20 ED visit ratings; two did not contribute. All participating physicians were offered a second round of ED visits for review, of which six agreed and completed. Overall, six physicians judged 40 ED visits each, and four physicians judged 20 ED visits each. ED physicians were provided the ED visits unique medical reporting number (MRN), which they used to retrieve the chart from the hospital’s electronic database. The physicians were blinded to the knowledge of the EDAC criteria and EDAC class to which an ED visit belonged. Following the chart review, the ED physicians answered two study questions (described below). The ED charts format, information and presentation were not altered in any way for the study. Each ED visit was rated by two ED physicians independently. We calculated a priori that 126 ED visits were needed to sufficiently power the study at an optimal 80% to detect statistical significance using a two tailed 0.05 alpha [32].

### Outcome measurement

Physicians answered two study questions based on their analysis of the ED visit. First, physicians were requested to judge whether an ED visit could have been appropriately and safely managed in a subacute primary care model. Second, we asked physicians to rate their confidence in this judgement using a 5-point Likert scale, ranging from not confident (1) to very confident (5) [34]. We provided the physicians with descriptions, definitions, staffing, diagnostic imaging and care services (i.e. laboratory, pharmaceutical) for various subacute centres to align understanding of subacute centre capabilities prior to providing ratings. Physicians received instruction and training on how to complete the questionnaire before the study commenced. All questionnaires were completed and submitted electronically using *CheckMarket* survey software.

### Statistical analysis

We reported demographic characteristics of the participating ED physicians and patient ED visits as frequencies and proportions. To determine if ED physicians could be established as a criterion standard, we calculated interrater reliability of physician agreement overall and for each EDAC class using a Cohen’s kappa coefficient with 95% confidence intervals (CIs). We predetermined a kappa coefficient equal to or greater than 0.6 would establish physician judgements as a criterion standard to identify avoidable ED visits [32]. This threshold was chosen as a 0.6 kappa indicated substantial agreement greater than chance [35]. Physician confidence scores were reported as means and standard deviations. We used a Spearman rank correlation to assess the correlation between the EDAC and ED physician judgements. Ordinal rankings of the EDAC were structured into three-levels of the classification (avoidable, potentially avoidable, not avoidable). Ordinal rankings of physician judgements were structured as: both physicians agreed the ED visit was only appropriate for the ED, the physicians disagreed (one judged the visit as only appropriate for the ED and the second judged as appropriate for subacute care), and both physicians agreed the ED visit was appropriate for subacute primary care. To understand the magnitude of association between the EDAC and ED physicians to classify avoidable ED visits, we computed a three-level ordinal logistic regression using odds ratios (ORs) and 95% CIs, along with the model’s area under the receiver operating characteristic curve (AUC). EDAC classes were modeled as a set of dummy variables with the not avoidable class set as the referent group. Finally, to determine the directionality of the potentially avoidable class towards either avoidable or not avoidable ED visits, we calculated the accuracy, sensitivity and specificity of the EDAC and ED physician judgements in three sequestered analyses. Initially, we computed baseline precision statistics using only avoidable and not avoidable ED visits. We repeated precision analyses when all potentially avoidable ED visits were classified as avoidable ED visits, then as not avoidable ED visits. We compared changes in precision measures relative to the initial analysis. Data were managed and analyzed using the ‘*dplyr’* package in R software(v. 3.6) [36].

### Ethics approval

Our study was approved by the Hamilton Integrated Research Ethics Board (HiREB), review reference number 2022-14625-GRA. Informed and written consent was obtained from all study participants.

## Results

### Characteristics of participating ED physicians

The participating physicians were mostly male (8), currently practicing in both the ED and an urgent care centre (8), and holding an academic appointment at a Canadian University (9). Physicians were nearly equal in medical training for the disciplines of emergency medicine (Fellow of The Royal College of Physicians of Canada; 6) and family medicine with emergency medicine certification (Certification in the College of Family Medicine, with Special Competence in Emergency Medicine; 4).

### Main results

Overall, there were 160 ED visits judged twice by different ED physicians, amassing 320 ratings. All ED visits were judged by two physicians; no visit was excluded, and no visit was reviewed only once. *Table 2* shows descriptive statistics of all ED visits used in the study and by EDAC class. The ED visits were fairly evenly distributed by sex (48% male, 52% female). ED visits were predominantly aged 18 to 40 years (48%), with 41 to 60 (31%) and 61 to 105 (21%) constituting the remainder. Visits were mostly assigned an urgent triage score (48%). Most visits had an ED physician recorded as the most responsible provider (81%) and were discharged from the ED (79%). The quantity of ED visits in each EDAC class were largely consistent (54, 53, and 53, respectively).

**Table 2:** Clinical and non-clinical characteristics of ED visits used in the study, categorized by EDAC class.

Characteristics	All ED Visits, n (%)	Avoidable ED Visits, n (%)	Potentially Avoidable ED Visits, n (%)	Not Avoidable ED Visits, n (%)
**Total ED Visits**	160	54	53	53
**Sex**				
Male	77 (48)	26 (48)	25 (47)	26 (49)
Female	83 (52)	28 (52)	28 (53)	27 (51)
**Age**, years				
18–40	77 (48)	31 (57)	31 (58)	15 (28)
41–60	49 (31)	18 (33)	22 (42)	5 (9)
61–105	34 (21)	5 (9)	0 (0)	33 (62)
**Mode of Arrival**				
Walk-In	109 (68)	47 (87)	36 (68)	26 (49)
Ambulance	51 (32)	7 (13)	17 (32)	27 (51)
**Triage Acuity**, CTAS				
1 –Resuscitation	2 (1)	0 (0)	0 (0)	2 (4)
2 –Emergent	23 (14)	0 (0)	0 (0)	23 (43)
3 –Urgent	77 (48)	0 (0)	53 (100)	24 (45)
4 –Less Urgent	41 (26)	38 (70)	0 (0)	3 (6)
5 –Non-Urgent	17 (11)	16 (30)	0 (0)	1 (2)
**Day of Week**				
Monday	25 (16)	7 (13)	7 (13)	11 (21)
Tuesday	23 (14)	6 (11)	9 (17)	8 (15)
Wednesday	28 (18)	11 (20)	6 (11)	11 (21)
Thursday	20 (13)	8 (15)	6 (11)	6 (11)
Friday	18 (11)	5 (9)	7 (13)	6 (11)
Saturday	20 (13)	9 (17)	6 (11)	5 (9)
Sunday	26 (16)	8 (15)	12 (23)	6 (11)
**Time in ED**^a^, minutes				
Average (SD)	429 (547)	213 (139)	238 (131)	839 (788)
Under 30	1 (1)	0 (0)	1 (2)	0 (0)
31–60	5 (3)	2 (4)	3 (6)	0 (0)
61–120	18 (11)	8 (15)	8 (15)	2 (4)
121–180	29 (18)	16 (30)	10 (19)	3 (6)
181–240	18 (11)	11 (20)	6 (11)	1 (2)
Over 241	89 (54)	15 (28)	24 (45)	47 (89)
**Most Responsible Provider**				
Emergency Medicine	129 (81)	54 (100)	53 (100)	22 (42)
Psychiatry	10 (6)	0 (0)	0 (0)	10 (19)
Internal Medicine	9 (6)	0 (0)	0 (0)	9 (17)
Nephrology	2 (1)	0 (0)	0 (0)	2 (4)
Cardiology	2 (1)	0 (0)	0 (0)	2 (4)
General Surgery	3 (2)	0 (0)	0 (0)	3 (6)
Other	5 (3)	0 (0)	0 (0)	5 (8)
**ED Visit Outcome**				
Discharged	127 (79)	54 (100)	53 (100)	20 (38)
Admission	27 (17)	0 (0)	0 (0)	27 (51)
Other	6 (4)	0 (0)	0 (0)	6 (11)

CTAS = Canadian Triage and Acuity Scale, SD = Standard Deviation.

^a^ Difference of triage time to left ED time; mean.

*Table 3* shows agreement between ED physicians after evaluating the ED visits. Physicians agreed on 139 (86.9%) of 160 ED visits, yielding a kappa coefficient of 0.69 (95% CI 0.59–0.79). Avoidable ED visits showed near-perfect agreement with 53 of 54 visits (98.1%). Potentially avoidable ED visits had the lowest agreement, 40 of 53 visits (75.5%), and the lowest kappa of 0.25 (95% CI 0.01–0.48). Not avoidable ED visits resulted in the highest kappa (0.70, 95% CI 0.53–0.87). Physician confidence scores mirrored the kappa gradient observed, giving the highest confidence in not avoidable ED visits and the lowest in potentially avoidable ED visits. ED visits identified as either avoidable or not avoidable were chosen to analyze ED visits strictly avoidable or not, yielding an almost agreement amongst physicians with a kappa of 0.84 (95% CI 0.73–0.95).

**Table 3:** Agreement between independent emergency physician’s judgments on the suitability of ED visits that could have been managed in subacute primary care.

	All ED Visits, n (%)	Avoidable and Not Avoidable ED Visits, n (%)	Avoidable ED visit, n (%)	Potentially Avoidable ED Visits, n (%)	Not Avoidable ED visits, n (%)
**Overall Agreement**	139 (86.9)	99 (92.5)	53 (98.1)	40 (75.5)	46 (86.8)
**Kappa**	0.69 (0.59–0.79)	0.84 (0.73–0.95)	0.66 (0.14–1.00)	0.25 (0.01–0.48)	0.70 (0.53–0.87)
**Kappa Interpretation**	Substantial agreement	Almost perfect agreement	Substantial agreement	Slight agreement	Substantial agreement
**Physician confidence,** mean (SD)	4.1 (0.9)	4.3 (0.9)	4.2 (0.9)	3.9 (0.9)	4.4 (0.8)

**Fig 1:**
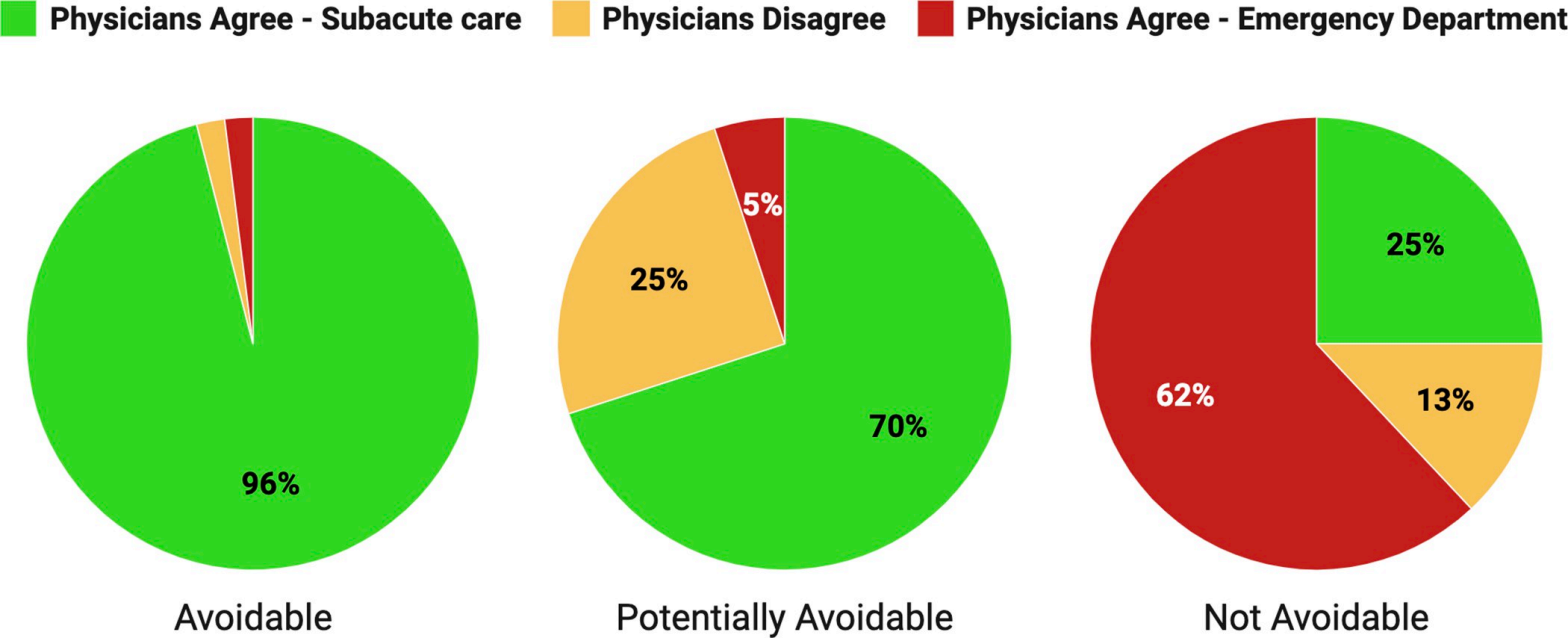
EDAC classes displaying the proportion of physician agreement of 160 ED visits.

*Fig 1* shows the proportion of physician agreement within each class of the EDAC. Of ED visits classified by the EDAC as avoidable, physicians agreed 52 (96.2%) were appropriate for management in subacute primary care. Of ED visits identified as potentially avoidable, physicians agreed 37 (69.8%) were suitable for subacute primary care, 13 (24.5%) were not suitable, and 3 (5.7%) had disagreement between physicians. Of ED visits classified as not avoidable from ED care, physicians agreed the ED was the appropriate centre on 33 visits (62.3%), 13 visits physicians agreed were appropriate for subacute care (24.5%), and 7 had physician disagreement (13.2%).

*Table 4* shows the results of the correlation and regression validity analyses. A Spearman rank correlation coefficient analysis found a significant association between the EDAC and ED physician judgement with a high and positive correlation coefficient (0.64; p<0.01). In an ordinal logistic regression analysis, the odds ED physicians agreed an ED visit was avoidable increased by 80 times when the EDAC identified an ED visit was avoidable (OR 80, 95% CI 17.1–374.9). The AUC of the regression model was 0.84, inferring this model is an excellent classifier to identify avoidable ED visits in comparison to physician agreement [37].

**Table 4:** Correlation and odds of ED physician agreement across EDAC classes using 160 ED visits from an academic hospital between January 1, 2019 to December 31, 2019.

Statistical Test	Result	p-value
**Spearman Rank Correlation**, coefficient	0.64	<0.01
**Ordinal Logistic Regression**, odds ratio (95% CI)		
Avoidable ED visits	80.0 (17.1–374.9)	<0.01
Potentially Avoidable ED visits	7.1 (3.0–16.8)	<0.01
Not Avoidable ED visits	-	-
Model AUC	0.84 (0.82–0.86)	

Note: CI = confidence interval, AUC = area under the receiver operator characteristic curve.

We conducted a sequestered analysis that examined whether the potentially avoidable class was more comparable to either the avoidable or not avoidable EDAC class. *Table 5* shows the results and *S2 Table* shows the raw data of the analyses. When including only avoidable and not avoidable visits, the EDAC showed strong accuracy (83.2%, 95% CI 77.5–88.0%) to delineate ED visits suitable for subacute care with high specificity (97.2%, 95% CI 92.1–99.4), and moderate sensitivity of (68.9%, 95% CI 59.1–77.3). When potentially avoidable ED visits were grouped with avoidable ED visits, accuracy and sensitivity remained relative consistent while specificity lowered 7.5%. When potentially avoidable ED visits were grouped with not avoidable ED visits, accuracy and sensitivity lowered substantially (21.6%, 25.5% respectively) while specificity remained constant.

**Table 5:** Accuracy, sensitivity and specificity of the EDAC’s capacity to identify ED visits avoidable, potentially avoidable and not avoidable against 320 ED physician judgements of 160 ED visits.

Classification of Potentially Avoidable Visits	Accuracy, % (95% CI)	Sensitivity, % (95% CI)	Specificity, % (95% CI)
Potentially Avoidable ED Visits Excluded	83.2 (77.5–88.0)	68.9 (59.1–77.3)	97.2 (92.1–99.4)
Potentially Avoidable ED Visits Classified as Avoidable	82.8 (78.2–86.8)	68.9 (59.1–77.5)	89.7 (84.9–93.4)
Potentially Avoidable ED Visits Classified as Not Avoidable	61.6 (56.0–66.9)	43.4 (36.6–50.4)	97.2 (92.1–99.4)

## Discussion

We successfully established ED physician judgment as a criterion standard for identifying ED visits that were suitable for subacute primary care. The EDAC demonstrated its validity as an accurate classifier of avoidable ED visits with a strong correlation and association to ED physician judgements. The EDAC exhibited high specificity, high accuracy and modest sensitivity, evidence of validity that substantiates its capacity to identify avoidable ED visits with precision in routinely collected ED data.

Our study further supports the EDAC as a valid classification tool. While previously published classifications such as ambulatory care sensitive conditions, family practice sensitive conditions, sentinel nonurgent conditions, and CTAS may offer more simplistic inclusion criteria compared to the EDAC, they may yield less accurate estimates of avoidable ED visits due to their limited reliance on one or two criteria variables [26, 38–40]. For example, ambulatory care-sensitive conditions and family practice-sensitive conditions rely solely on diagnostic criteria to define their cohorts, a characteristic our previous consensus study found to be isolated from identifying avoidable ED visits [26, 33, 38, 40]. Most published classifications lack validity testing to a criterion standard, a critical phase needed to determine a classifications generalizability [26, 32, 33, 39]. We speculate that researchers have relied on these classifications due to convenience and insufficient information regarding their validation, a gap in the literature this study aimed to address with the EDAC.

Practically, the EDAC could serve as a valuable measure for policymakers seeking to address ED overcrowding, optimize healthcare resource utilization, and improve care continuity in primary care. The EDAC has the potential to augment or enhance existing quality indicators of ED use, providing a clearer understanding of patient cohorts using the ED improperly when non-ED care is capable of managing their primary condition [26, 39]. The EDAC could plausibly be leveraged as an indirect indicator of access to primary care and the healthcare system’s capacity to manage patients with primary care conditions, thus aiding in understanding patient trends in ED use for primary care treatable conditions. Epidemiologically, the EDAC could support the investigation of neighbourhood characteristics that influence avoidable ED visits. Understanding these characteristics could inform intervention development to address geospatial care gaps [41]. Lastly, the EDAC could guide interventions promoting integration with primary care, virtual care, or new care models (i.e., paramedic transport to non-ED care centres) [42, 43].

The EDAC was constructed with a conservative approach towards identifying avoidable ED visits, as evidenced with its high specificity [30, 33]. The low proportion of avoidable and potentially avoidable ED visits judged by ED physicians as requiring ED care further supports it as a highly specific classifier for visits that could have been managed outside of the ED. We attribute our results to the inclusion of the main ED physician intervention as a criterion, a variable not commonly incorporated in previous epidemiological classifications [30]. Based on our study’s experimental methodology and results, we assert the EDAC’s performance can be replicated in other EDs seeking to identify and understand avoidable ED visits.

An important finding of our study was gaining insights into the potentially avoidable class in the EDAC. When grouped with avoidable ED visits, specificity decreased marginally while accuracy remained consistent. When grouped with not avoidable visits, specificity remained constant, but accuracy decreased substantially. These results, combined with approximately 70% of ED physicians agreeing that potentially avoidable visits were suitable for subacute care, suggest that potentially avoidable visits align more closely with avoidable visits than not avoidable visits.

A critical feature of our study was establishing ED physician judgement as a reliable criterion in the absence of a gold standard [32, 35]. Overall, substantial agreement was observed among ED physicians, with near perfect agreement when evaluating only avoidable and not avoidable ED visits. These results, combined with their confidence scores, support our a priori hypothesis that ED physicians possess a strong and similar clinical understanding of which ED visits require ED medicine, ED services or general hospital care, and which visits do not [32]. Potentially avoidable visits showed slight agreement among ED physicians, a result consistent with the theoretical nature of a middle-level category within a classification [44].

Although the EDAC demonstrated criterion validity, the EDAC is not a pragmatic tool for clinical decision-making at the time of care, since it is based on post-ED visit information (i.e., ED discharge). However, the EDAC can be used for epidemiological comparisons, benchmarking, and program evaluations, where the desired outcome of the model is to classify avoidable ED visits.

### Limitations

Physicians could not be blinded to patient identity, potentially introducing implicit bias if they were familiar with the ED visit [45]. However, this is unlikely given the very low probability that a participating physician encountered the ED visit and could recount the visit several years previously. We contend this bias was minimal due to the incorporation of randomization in selection of ED visits and random assignment to each physician. Though we established physician judgment as a criterion standard in our analyses, we acknowledge that the same interrater agreement may vary in different EDs. While ten ED physicians were deemed sufficient for conducting this study, recruiting additional physicians could have enhanced the robustness of the results. Future research could reproduce this study with more study centres (academic hospitals), ED physicians and ED visits to further validate the EDAC. Physician agreement supplied a benchmark for comparison with the EDAC, although this is not a gold standard. Lastly, the EDAC provides a broad classification of ED visits that could have been managed in non-ED settings but cannot discern the necessity of a visit for different population groups, such as marginalized patients, patients of lower socioeconomic status, or patients facing disproportionate barriers to primary care.

### Conclusion

The Emergency Department Avoidability Classification demonstrated evidence of validity against ED physician judgements as a trustworthy retrospective classifier of avoidable ED visits. This classification has potential to inform epidemiological examinations of avoidable ED visits, support the development of ED avoidable models, triaging tools and as an outcome indicator for experimentation and benchmarking. Pragmatically, the EDAC could permit cohort and geospatial analyses to improve our understanding of community care gaps that may contribute to avoidable ED visits, and subsequently ED overcrowding.

## Supporting information

S1 Table List of canadian classification of health interventions included in the EDAC. Intervention Codes for Avoidable and Potentially Avoidable classes of the EDAC.
(DOCX)

S2 Table Contingency tables. Data of EDAC classes and ED physician judgments with different groupings of the Potentially Avoidable class.
(DOCX)

## References

[1] Lowthian JA, Curtis AJ, Cameron PA, Stoelwinder JU, Cooke MW, McNeil JJ. Systematic review of trends in emergency department attendances: an Australian perspective. Emerg Med J [Internet]. 2011 May 1 [cited 2023 Sep 10];28(5):373–7. Available from: https://emj.bmj.com/content/28/5/373 20961936 10.1136/emj.2010.099226

[2] Morganti KG, Bauhoff S, Blanchard JC, Abir M, Iyer N, Smith A, et al. The Evolving Role of Emergency Departments in the United States. Rand Health Q [Internet]. 2013 Jun 1 [cited 2023 Apr 4];3(2):3. Available from: https://www.ncbi.nlm.nih.gov/pmc/articles/PMC4945168/ 28083290 10.7249/rhq-03-02-03 PMC4945168

[3] Hsia RY, Zagorov S, Sarkar N, Savides MT, Feldmeier M, Addo N. Patterns in Patient Encounters and Emergency Department Capacity in California, 2011–2021. JAMA Netw Open [Internet]. 2023 Jun 22 [cited 2023 Sep 10];6(6):e2319438. Available from: doi: 10.1001/jamanetworkopen.2023.19438 37347481 PMC10288334

[4] Strum RP, Drennan IR, Mowbray FI, Mondoux S, Worster A, Babe G, et al. Increased demand for paramedic transports to the emergency department in Ontario, Canada: a population-level descriptive study from 2010 to 2019. Can J Emerg Med [Internet]. 2022 Aug 19 [cited 2022 Aug 24]; Available from: 10.1007/s43678-022-00363-4 PMC9389513 35984572

[5] Derlet RW. Overcrowding in emergency departments: increased demand and decreased capacity. Ann Emerg Med. 2002 Apr;39(4):430–2. 11919530 10.1067/mem.2002.122707

[6] Savioli G, Ceresa IF, Gri N, Bavestrello Piccini G, Longhitano Y, Zanza C, et al. Emergency Department Overcrowding: Understanding the Factors to Find Corresponding Solutions. J Pers Med [Internet]. 2022 Feb 14 [cited 2023 May 5];12(2):279. Available from: https://www.ncbi.nlm.nih.gov/pmc/articles/PMC8877301/ doi: 10.3390/jpm12020279 35207769 PMC8877301

[7] Pines JM, Pollack CV, Diercks DB, Chang AM, Shofer FS, Hollander JE. The association between emergency department crowding and adverse cardiovascular outcomes in patients with chest pain. Acad Emerg Med Off J Soc Acad Emerg Med. 2009 Jul;16(7):617–25. doi: 10.1111/j.1553-2712.2009.00456.x 19549010

[8] Pines JM, Hollander JE. Emergency department crowding is associated with poor care for patients with severe pain. Ann Emerg Med. 2008 Jan;51(1):1–5. doi: 10.1016/j.annemergmed.2007.07.008 17913299

[9] Trzeciak S, Rivers EP. Emergency department overcrowding in the United States: an emerging threat to patient safety and public health. Emerg Med J EMJ. 2003 Sep;20(5):402–5. doi: 10.1136/emj.20.5.402 12954674 PMC1726173

[10] Chalfin DB, Trzeciak S, Likourezos A, Baumann BM, Dellinger RP, DELAY-ED study group. Impact of delayed transfer of critically ill patients from the emergency department to the intensive care unit. Crit Care Med. 2007 Jun;35(6):1477–83. 17440421 10.1097/01.CCM.0000266585.74905.5A

[11] Richardson DB. Increase in patient mortality at 10 days associated with emergency department overcrowding. Med J Aust. 2006 Mar 6;184(5):213–6. doi: 10.5694/j.1326-5377.2006.tb00204.x 16515430

[12] Kulstad EB, Sikka R, Sweis RT, Kelley KM, Rzechula KH. ED overcrowding is associated with an increased frequency of medication errors. Am J Emerg Med. 2010 Mar;28(3):304–9. doi: 10.1016/j.ajem.2008.12.014 20223387

[13] Shin TG, Jo IJ, Choi DJ, Kang MJ, Jeon K, Suh GY, et al. The adverse effect of emergency department crowding on compliance with the resuscitation bundle in the management of severe sepsis and septic shock. Crit Care [Internet]. 2013 Oct 6 [cited 2023 May 5];17(5):R224. Available from: doi: 10.1186/cc13047 24093643 PMC4055965

[14] Diercks DB, Roe MT, Chen AY, Peacock WF, Kirk JD, Pollack CV, et al. Prolonged Emergency Department Stays of Non–ST-Segment-Elevation Myocardial Infarction Patients Are Associated With Worse Adherence to the American College of Cardiology/American Heart Association Guidelines for Management and Increased Adverse Events. Ann Emerg Med [Internet]. 2007 Nov 1 [cited 2023 May 5];50(5):489–96. Available from: https://www.sciencedirect.com/science/article/pii/S0196064407004453 doi: 10.1016/j.annemergmed.2007.03.033 17583379

[15] Tekwani KL, Kerem Y, Mistry CD, Sayger BM, Kulstad EB. Emergency Department Crowding is Associated with Reduced Satisfaction Scores in Patients Discharged from the Emergency Department. West J Emerg Med Integrating Emerg Care Popul Health [Internet]. 2013 [cited 2023 May 5];14(1). Available from: https://escholarship.org/uc/item/5ks6092b doi: 10.5811/westjem.2011.11.11456 23447751 PMC3582517

[16] Huang Q, Thind A, Dreyer JF, Zaric GS. The impact of delays to admission from the emergency department on inpatient outcomes. BMC Emerg Med [Internet]. 2010 Jul 9 [cited 2023 Apr 26];10:16. Available from: https://www.ncbi.nlm.nih.gov/pmc/articles/PMC2912828/ doi: 10.1186/1471-227X-10-16 20618934 PMC2912828

[17] Kim D uk Park YS, Park JM Brown NJ, Chu K Lee JH, et al. Influence of Overcrowding in the Emergency Department on Return Visit within 72 H. J Clin Med [Internet]. 2020 May 9 [cited 2023 Sep 10];9(5):1406. Available from: https://www.ncbi.nlm.nih.gov/pmc/articles/PMC7290478/32397560 10.3390/jcm9051406 PMC7290478

[18] Affleck A, Parks P, Drummond A, Rowe BH, Ovens HJ. Emergency department overcrowding and access block. Can J Emerg Med [Internet]. 2013 Nov [cited 2021 Dec 29];15(6):359–70. Available from: https://www.cambridge.org/core/journals/canadian-journal-of-emergency-medicine/article/emergency-department-overcrowding-and-access-block/4C6FD86D8E427379D53EEEDB0B58FB43 doi: 10.1017/s1481803500002451 24176460

[19] Oredsson S, Jonsson H, Rognes J, Lind L, Göransson KE, Ehrenberg A, et al. A systematic review of triage-related interventions to improve patient flow in emergency departments. Scand J Trauma Resusc Emerg Med [Internet]. 2011 Jul 19 [cited 2023 Apr 26];19:43. Available from: https://www.ncbi.nlm.nih.gov/pmc/articles/PMC3152510/ doi: 10.1186/1757-7241-19-43 21771339 PMC3152510

[20] Feral-Pierssens AL, Morris J, Marquis M, Daoust R, Cournoyer A, Lessard J, et al. Safety assessment of a redirection program using an electronic application for low-acuity patients visiting an emergency department. BMC Emerg Med [Internet]. 2022 Apr 29 [cited 2023 May 10];22(1):71. Available from: doi: 10.1186/s12873-022-00626-4 35488215 PMC9052637

[21] Morin C, Choukroun J, Callahan JC. Safety and efficiency of a redirection procedure toward an out of hours general practice before admission to an emergency department, an observational study. BMC Emerg Med [Internet]. 2018 Aug 22 [cited 2023 May 10];18(1):26. Available from: doi: 10.1186/s12873-018-0173-6 30134934 PMC6103978

[22] Gaeta S, Edwards T, Bourenane S, Gonzalez CE, McFarland K, Milling D. Emergency department surge and overcrowding: An interdisciplinary solution for an institutional issue. J Clin Oncol [Internet]. 2019 Sep 20 [cited 2023 Feb 23];37(27_suppl):242–242. Available from: https://ascopubs.org/doi/abs/10.1200/JCO.2019.37.27_suppl.242

[23] Alishahi Tabriz A, Turner K, Hong YR, Gheytasvand S, Powers BD, Elston Lafata J. Trends and Characteristics of Potentially Preventable Emergency Department Visits Among Patients With Cancer in the US. JAMA Netw Open [Internet]. 2023 Jan 19 [cited 2023 Feb 23];6(1):e2250423. Available from: doi: 10.1001/jamanetworkopen.2022.50423 36656584 PMC9857289

[24] Lasserson DS, Harris C, Elias T, Bowen J, Clare S. What is the evidence base for ambulatory care for acute medical illness? Acute Med. 2018;17(3):148–53. 30129948

[25] Parkinson B, Meacock R, Checkland K, Sutton M. Clarifying the concept of avoidable emergency department attendance. J Health Serv Res Policy [Internet]. 2021 Jan 1 [cited 2023 Apr 26];26(1):68–73. Available from: doi: 10.1177/1355819620921894 32517553 PMC7734604

[26] Lau T, Maltby A, Ali S, Moran V, Wilk P. Does the definition of preventable emergency department visit matter? An empirical analysis using 20 million visits in Ontario and Alberta. Acad Emerg Med [Internet]. 2022 [cited 2023 Feb 7];29(11):1329–37. Available from: http://onlinelibrary.wiley.com/doi/abs/10.1111/acem.14587 36043233 10.1111/acem.14587

[27] Hsia RY, Niedzwiecki M. Avoidable emergency department visits: a starting point. Int J Qual Health Care [Internet]. 2017 Oct 1 [cited 2023 Mar 10];29(5):642–5. Available from: doi: 10.1093/intqhc/mzx081 28992158

[28] Moskop JC, Sklar DP, Geiderman JM, Schears RM, Bookman KJ. Emergency department crowding, part 1—concept, causes, and moral consequences. Ann Emerg Med. 2009 May;53(5):605–11. doi: 10.1016/j.annemergmed.2008.09.019 19027193

[29] Strum RP, Tavares W, Worster A, Griffith LE, Rahim A, Costa AP. Development of the PriCARE classification for potentially preventable emergency department visits by ambulance: a RAND/UCLA modified Delphi study protocol. BMJ Open. 2021 Jan 20;11(1):e045351. doi: 10.1136/bmjopen-2020-045351 33472792 PMC7818828

[30] Strum RP, Tavares W, Worster A, Griffith LE, Costa AP. Emergency department interventions that could be conducted in subacute care settings for patients with nonemergent conditions transported by paramedics: a modified Delphi study. Can Med Assoc Open Access J [Internet]. 2022 Jan 1 [cited 2022 Jan 11];10(1):E1–7. Available from: https://www.cmajopen.ca/content/10/1/E1 doi: 10.9778/cmajo.20210148 35017171 PMC8758169

[31] Strum RP, Tavares W, Worster A, Griffith LE, Costa AP. Identifying patient characteristics associated with potentially redirectable paramedic transported emergency department visits in Ontario, Canada: a population-based cohort study. BMJ Open [Internet]. 2021 Dec 1 [cited 2021 Dec 31];11(12):e054625. Available from: https://bmjopen.bmj.com/content/11/12/e054625 doi: 10.1136/bmjopen-2021-054625 35225823 PMC8718420

[32] Strum RP, Mondoux S, Mowbray F, Worster A, Griffith LE, Tavares W, et al. Validation of a classification to identify emergency department visits suitable for subacute and virtual care models: a randomised single-blinded agreement study protocol. BMJ Open [Internet]. 2022 Dec 1 [cited 2023 Jan 19];12(12):e068488. Available from: https://bmjopen.bmj.com/content/12/12/e068488 10.1136/bmjopen-2022-068488 PMC9764606 36526315

[33] Strum RP, Tavares W, Worster A, Griffith LE, Costa AP. Inclusion of patient-level emergency department characteristics to classify potentially redirectable visits to subacute care: a modified Delphi consensus study. CMAJ Open [Internet]. 2023 Jan [cited 2023 Feb 7];11(1):E70–6. Available from: http://cmajopen.ca/lookup/doi/10.9778/cmajo.20220062 36693658 10.9778/cmajo.20220062 PMC9876581

[34] Sullivan GM, Artino AR. Analyzing and Interpreting Data From Likert-Type Scales. J Grad Med Educ [Internet]. 2013 Dec [cited 2020 Jul 8];5(4):541–2. Available from: http://www.jgme.org/doi/abs/10.4300/JGME-5-4-18 24454995 10.4300/JGME-5-4-18 PMC3886444

[35] McHugh ML. Interrater reliability: the kappa statistic. Biochem Medica [Internet]. 2012 Oct 15 [cited 2022 Jan 7];22(3):276–82. Available from: https://www.ncbi.nlm.nih.gov/pmc/articles/PMC3900052/ doi: 10.1016/j.jocd.2012.03.005 23092060 PMC3900052

[36] Wickham H, François R, Henry L, Müller K, RStudio. dplyr: A Grammar of Data Manipulation [Internet]. 2021 [cited 2022 Jan 23]. Available from: https://CRAN.R-project.org/package=dplyr

[37] Jr DWH, Lemeshow S, Sturdivant RX. Applied Logistic Regression. John Wiley & Sons; 2013. 528 p.

[38] Frick J, Möckel M, Muller R, Searle J, Somasundaram R, Slagman A. Suitability of current definitions of ambulatory care sensitive conditions for research in emergency department patients: a secondary health data analysis. BMJ Open. 2017 Oct 22;7(10):e016109. doi: 10.1136/bmjopen-2017-016109 29061605 PMC5665266

[39] van der Pol M, Olajide D, Dusheiko M, Elliott R, Guthrie B, Jorm L, et al. The impact of quality and accessibility of primary care on emergency admissions for a range of chronic ambulatory care sensitive conditions (ACSCs) in Scotland: longitudinal analysis. BMC Fam Pract [Internet]. 2019 Dec [cited 2021 Oct 12];20(1):32. Available from: https://bmcfampract.biomedcentral.com/articles/10.1186/s12875-019-0921-z 30795737 10.1186/s12875-019-0921-z PMC6385424

[40] Alberta Health Services. Family Practice Sensitive Conditions. Gov Alta. 2011;8.

[41] Samarasundera E, Martin D, Saxena S, Majeed A. Socio-demographic data sources for monitoring locality health profiles and geographical planning of primary health care in the UK. Prim Health Care Res Dev [Internet]. 2010 Oct [cited 2024 Jan 7];11(4):287–300. Available from: https://www.cambridge.org/core/journals/primary-health-care-research-and-development/article/sociodemographic-data-sources-for-monitoring-locality-health-profiles-and-geographical-planning-of-primary-health-care-in-the-uk/E8CA175BE2450E71D3C167955C4AF93A

[42] McFadzean IJ, Edwards M, Davies F, Cooper A, Price D, Carson-Stevens A, et al. Realist analysis of whether emergency departments with primary care services generate ‘provider-induced demand.’ BMC Emerg Med [Internet]. 2022 Sep 6 [cited 2024 Jan 7];22(1):155. Available from: doi: 10.1186/s12873-022-00709-2 36068508 PMC9450363

[43] Kiran T, Wang R, Handford C, Laraya N, Eissa A, Pariser P, et al. Family physician practice patterns during COVID-19 and future intentions: Cross-sectional survey in Ontario, Canada. Can Fam Physician [Internet]. 2022 Nov 1 [cited 2023 Apr 26];68(11):836–46. Available from: https://www.cfp.ca/content/68/11/836 doi: 10.46747/cfp.6811836 36376032 PMC9833162

[44] Hallgren KA. Computing Inter-Rater Reliability for Observational Data: An Overview and Tutorial. Tutor Quant Methods Psychol [Internet]. 2012 [cited 2024 Jan 7];8(1):23–34. Available from: https://www.ncbi.nlm.nih.gov/pmc/articles/PMC3402032/ doi: 10.20982/tqmp.08.1.p023 22833776 PMC3402032

[45] Karanicolas PJ, Farrokhyar F, Bhandari M. Blinding: Who, what, when, why, how? Can J Surg [Internet]. 2010 Oct [cited 2024 Jan 7];53(5):345–8. Available from: https://www.ncbi.nlm.nih.gov/pmc/articles/PMC2947122/20858381 PMC2947122

