# Supplementary material for: Validating the Emergency Department Avoidability Classification (EDAC): A cluster randomized single-blinded agreement study
Source: PLoS One. 2024 Jan 23;19(1):e0297689. doi: 10.1371/journal.pone.0297689 (PMC10805301; doi:10.1371/journal.pone.0297689)
Supplement: S1 Table — Intervention Codes for Avoidable and Potentially Avoidable classes of the EDAC. (DOCX) [file pone.0297689.s001.docx]

**S1 Table:** List of Canadian Classification of Health Interventions included in the EDAC for Avoidable and Potentially Avoidable classes.

| **Canadian Classification of Health Interventions Section** | **Physician Intervention** | **Codification** |
| --- | --- | --- |
| (1) Physical/ Physiological Therapeutic Interventions | Control of bleeding, nose using per orifice approach and agent NEC [e.g. silver nitrate] | 1ET13CAZ9 |
|  | Control of bleeding, nose using per orifice approach and device NEC (e.g. electrocautery) | 1ET13CAGX |
|  | Control of bleeding, nose using per orifice approach and packing | 1ET13CANP |
|  | Drainage, bladder using per orifice approach and drainage catheter | 1PM52CATS |
|  | Extraction, rectum using per orifice approach and manual technique | 1NQ57CJ |
|  | Immobilization, knee joint using splinting device [e.g. supportive and corrective] | 1VG03JASR |
|  | Immobilization, shoulder joint using sling | 1TA03JASQ |
|  | Implantation of internal device, stomach of gastric tube [e.g. nasogastric feeding tube] using per orifice approach | 1NF53CATS |
|  | Implantation of internal device, vein NEC of intravenous catheter using percutaneous approach | 1KX53HAFT |
|  | Management of internal device, bladder of catheter using per orifice approach | 1PM54CATS |
|  | Management of internal device, stomach of percutaneously inserted gastric tube [PEG] | 1NF54HATS |
|  | Oxygenation, respiratory system NEC using bulk storage manifold system | 1GZ32CAMY |
|  | Pharmacotherapy (local), circulatory system NEC percutaneous infusion approach of electrolyte balance agents | 1LZ35HHC7 |
|  | Pharmacotherapy (local), rectum using per orifice approach and agent NEC (e.g. oil retention, soap suds) | 1NQ35CAZ9 |
|  | Pharmacotherapy (local), respiratory system NEC using antiasthmatic agent | 1GZ35CAR3 |
|  | Pharmacotherapy, total body blood and blood forming organ agents percutaneous approach [intramuscular, intravenous, subcutaneous, intradermal] using antithrombotic agent | 1ZZ35HAC1 |
|  | Pharmacotherapy, total body general antiinfective agents percutaneous approach [intramuscular, intravenous, subcutaneous, intradermal] cephalosporin and related substance | 1ZZ35HAK4 |
|  | Pharmacotherapy, total body musculoskeletal system agents percutaneous approach [intramuscular, intravenous, subcutaneous, intradermal] antiinflammatory and antirheumatic agent | 1ZZ35HAN1 |
|  | Pharmacotherapy, total body nervous system agents percutaneous approach [intramuscular, intravenous, subcutaneous, intradermal] analgesic | 1ZZ35HAP2 |
|  | Reduction, small and large intestine using manual technique (for hernia reduction alone) | 1NP73JH |
|  | Reduction, wrist joint using closed (external) approach | 1UB73JA |
|  | Repair, lip using apposition technique [e.g. suture] | 1YE80LA |
|  | Repair, scalp using apposition technique [e.g. suture, staple] | 1YA80LA |
|  | Repair, scalp using closure device (e.g. clip, adhesive skin closure [Steri-Strips]) | 1YA80JAFF |
|  | Repair, scalp using glue for apposition (e.g. crazy glue, glustitch) | 1YA80LAW4 |
|  | Repair, skin of abdomen and trunk using open apposition technique [suture] | 1YS80LA |
|  | Repair, skin of arm using apposition technique [suture] | 1YT80LA |
|  | Repair, skin of arm using closure device (e.g. clip, adhesive skin closure [Steri-Strips]) | 1YT80JAFF |
|  | Repair, skin of ear using apposition technique [e.g. suture] | 1YC80LA |
|  | Repair, skin of face using apposition technique [suture] | 1YF80LA |
|  | Repair, skin of face using closure device (e.g. clip, adhesive skin closure [Steri-Strips]) | 1YF80JAFF |
|  | Repair, skin of face using glue for apposition (e.g. crazy glue or glustitch) | 1YF80LAW4 |
|  | Repair, skin of foot using apposition technique [suture] | 1YW80LA |
|  | Repair, skin of forehead using apposition technique [e.g. suturing, stapling] | 1YB80LA |
|  | Repair, skin of forehead using closure device (e.g.clip, adhesive skin closure [Steri-Strips]) | 1YB80JAFF |
|  | Repair, skin of forehead using glue (e.g. crazy glue, glustitch) | 1YB80LAW4 |
|  | Repair, skin of hand using apposition technique [suture] | 1YU80LA |
|  | Repair, skin of hand using closure device (e.g. clip, adhesive skin closure [Steri-Strips]) | 1YU80JAFF |
|  | Repair, skin of hand using glue for apposition (e.g. crazy glue, glustitch) | 1YU80LAW4 |
|  | Repair, skin of leg using apposition technique [suture] | 1YV80LA |
|  | Repair, skin of leg using closure device (e.g. clip, adhesive skin closure [Steri-Strips]) | 1YV80JAFF |
|  | Repair, skin of nose using apposition technique [e.g. suture] | 1YD80LA |
| (2) Diagnostic Interventions | Assessment (examination), total body general NEC (e.g. multiple reasons) | 2ZZ02ZZ |
|  | Electrophysiological measurement, heart NEC external application using recording electrodes (or ECG NOS) | 2HZ24JAXJ |
|  | Function study, bladder capacity determination | 2PM58VE |
|  | Function study, bladder post- void residual volume measurement | 2PM58VD |
|  | Function study, respiratory system at rest (steady state) | 2GZ58TA |
|  | Inspection, rectum using per orifice manual (digital) technique | 2NQ70CA |
|  | Specimen collection (for diagnostic testing), total body blood by venous puncture | 2ZZ13RA |
| (3) Diagnostic Imaging Interventions | Ultrasound, abdominal cavity alone | 3OT30DA |
|  | Ultrasound, abdominal cavity transvaginal probe | 3OT30LA |
|  | Ultrasound, arteries of leg NEC with Doppler | 3KG30DC |
|  | Ultrasound, bladder NOS alone | 3PM30DA |
|  | Ultrasound, female genital tract NEC alone | 3RZ30DA |
|  | Ultrasound, female genital tract NEC transvaginal approach | 3RZ30LA |
|  | Ultrasound, kidney alone | 3PC30DA |
|  | Ultrasound, leg NEC alone | 3VZ30DA |
|  | Ultrasound, scrotum alone | 3QG30DA |
|  | Ultrasound, thoracic cavity NEC alone | 3GY30DA |
|  | Ultrasound, veins of arm NEC with Doppler | 3JU30DC |
|  | Ultrasound, veins of leg NEC alone | 3KR30DA |
|  | Ultrasound, veins of leg NEC with color flow and Doppler | 3KR30DC |
|  | Ultrasound, veins of leg NEC with Doppler | 3KR30DD |
|  | Xray, abdominal cavity without contrast (with or without fluoroscopy) | 3OT10VA |
|  | Xray, ankle joint without contrast (e.g. plain film) (with or without fluoroscopy) | 3WA10VA |
|  | Xray, clavicle without contrast (with or without fluoroscopy) | 3SM10VA |
|  | Xray, elbow joint without contrast | 3TM10VA |
|  | Xray, facial bone structure without contrast (e.g. plain film) | 3EI10VA |
|  | Xray, femur without contrast (with or without fluoroscopy) | 3VC10VA |
|  | Xray, foot without contrast (e.g. plain film) (with or without fluoroscopy) | 3WG10VA |
|  | Xray, hand with wrist without contrast (e.g. plain film) (with or without fluoroscopy) | 3UZ10VA |
|  | Xray, hip joint without contrast (with or without fluoroscopy) | 3VA10VA |
|  | Xray, humerus without contrast (e.g. plain film) (with or without fluoroscopy) | 3TK10VA |
|  | Xray, joints of fingers and hand NEC without contrast (e.g. plain film) (with or without fluoroscopy) | 3UL10VA |
|  | Xray, kidney with ureter and bladder without contrast (e.g. plain film KUB) | 3PS10VA |
|  | Xray, knee joint without contrast (with or without fluoroscopy) | 3VG10VA |
|  | Xray, lung NEC without contrast (e.g. plain film) (with or without fluoroscopy) | 3GT10VA |
|  | Xray, mandible without contrast (e.g. plain film) (with or without fluoroscopy) | 3EE10VA |
|  | Xray, nose without contrast (e.g. plain film) (with or without fluoroscopy) | 3ET10VA |
|  | Xray, pelvis without contrast | 3SQ10VA |
|  | Xray, radius and ulna without contrast (e.g. plain film) (with or without fluoroscopy) | 3TV10VA |
|  | Xray, ribs without contrast (with or without fluoroscopy) | 3SL10VA |
|  | Xray, sacrum and coccyx without contrast | 3SF10VA |
|  | Xray, shoulder joint without contrast (with or without fluoroscopy) | 3TA10VA |
|  | Xray, soft tissue of head and neck without contrast (e.g. plain film) (with or without fluoroscopy) | 3EQ10VA |
|  | Xray, spinal vertebrae without contrast | 3SC10VA |
|  | Xray, sternum without contrast (with or without fluoroscopy) | 3SK10VA |
|  | Xray, thoracic cavity NEC without contrast (with or without fluoroscopy) | 3GY10VA |
|  | Xray, tibia and fibula without contrast (e.g. plain film) (with or without fluoroscopy) | 3VQ10VA |
|  | Xray, wrist joint without contrast (e.g. plain film) (with or without fluoroscopy) | 3UB10VA |
| (6) Cognitive, Psychosocial and Sensory Therapeutic Interventions | Assessment, mental health and addictions for capacity for harm (to self or others) | 6AA02CP |
|  | Assessment, mental health and addictions for coping skills NEC | 6AA02SK |
|  | Assessment, mental health and addictions for other reason NEC | 6AA02ZZ |
|  | Counseling, mental health for substance addiction | 6AA10AD |
|  | Counseling, mental health for behavior | 6AA10BE |
|  | Counseling, mental health and addictions for concurrent disorders | 6AA10CD |
|  | Counseling, mental health for trauma NEC | 6AA10CT |
|  | Counseling, mental health for mood (e.g. anger, anxiety, relaxation, leisure) | 6AA10MA |
|  | Counseling, mental health for other reasons | 6AA10ZZ |
|  | Therapy, mental health crisis/trauma active listening | 6AA30CTAA |
|  | Assessment, motor and living skills for activities of daily living [ADL] | 6VA02ZZ |
| (7) Other Healthcare Interventions | Counseling, promoting health and preventing disease for other reason | 7SP10ZZ |
| (8) Therapeutic Interventions Strengthening the Immune System | Immunization (to prevent) diphtheria and tetanus by intramuscular [IM] injection of toxoid | 8MK70HABK |
