# Supplementary material for: Validating the Emergency Department Avoidability Classification (EDAC): A cluster randomized single-blinded agreement study
Source: PLoS One. 2024 Jan 23;19(1):e0297689. doi: 10.1371/journal.pone.0297689 (PMC10805301; doi:10.1371/journal.pone.0297689)
Supplement: S2 Table — Data of EDAC classes and ED physician judgments with different groupings of the Potentially Avoidable class. (DOCX) [file pone.0297689.s002.docx]

**S2 Table:** Contingency tables of EDAC frequencies compared to ED physician judgments with different groupings of the potentially avoidable class.

| **Potentially Avoidable ED Visits Excluded** | | | |
| --- | --- | --- | --- |
|  | | **EDAC** | |
|  |  | Appropriate for ED Only | Appropriate for Subacute Primary Care |
| **ED Physicians** | Appropriate for ED Only | 73 | 3 |
|  | Appropriate for Subacute Primary Care | 33 | 105 |

| **Potentially Avoidable ED Visits Classified as Avoidable** | | | |
| --- | --- | --- | --- |
|  | | **EDAC** | |
|  |  | Appropriate for ED Only | Appropriate for Subacute Primary Care |
| **ED Physicians** | Appropriate for ED Only | 73 | 22 |
|  | Appropriate for Subacute Primary Care | 33 | 192 |

| **Potentially Avoidable ED Visits Classified as Not Avoidable** | | | |
| --- | --- | --- | --- |
|  | | **EDAC** | |
|  |  | Appropriate for ED Only | Appropriate for Subacute Primary Care |
| **ED Physicians** | Appropriate for ED Only | 92 | 3 |
|  | Appropriate for Subacute Primary Care | 120 | 105 |
